# The geography of risk: understanding disparities in nonmedical opioid mortality and the role of socio-built environments in New Jersey

**DOI:** 10.1186/s12954-025-01332-7

**Published:** 2026-02-21

**Authors:** Barbara Tempalski, Chunki Fong, Sean T. Doyle, Danielle C. Ompad

**Affiliations:** 1 Center for Community-Based Population Health Research, National Development & Research Institutes - USA (NDRI-USA), Inc., 31 West 34th Street, New York, NY 10001 USA; 2 NDRI-USA. Inc., Institute for Implementation Science in Population Health, CUNY Graduate School of Public Health & Health Policy, New York, NY 10027 USA; 3 NDRI-USA, Inc., Social Sciences Innovations Corporation, Colorado Springs, CO 80927 USA; 4 Center for Drug Use and HIV, HCV Research, New York University School of Global Public Health, 708 Broadway, New York, NY 10003 USA

**Keywords:** Nonmedical opioid mortality, Social and built environments, Housing instability, Urban–suburban–rural disparities, Opioid use–related health services

## Abstract

**Background:**

Disparities in nonmedical opioid (NMO) mortality reflect a shifting geography of risk that presents urgent public health challenges. This study uses a socio-built environment (SBE) framework to investigate how place-based conditions shape NMO-related risks across urban, suburban, and rural municipalities in New Jersey.

**Methods:**

Six SBE domains with multiple indicators were analyzed. Generalized linear models with a negative binomial distribution examined associations with NMO mortality, estimating incidence rate ratios with 95% confidence intervals. Domain-level contributions were assessed using log-likelihood ratio chi-square tests, with models stratified by geographies.

**Results:**

The quality of residential, commercial, and community economic engagement domains contributed significantly to NMO mortality across all municipalities. The physical environment, community participation, and spatial access to opioid health programs domains were more influential in urban settings, with weaker or inconsistent effects in suburban and rural areas. Foreclosure rates, vacant storefronts, liquor license density, and greater economic distress were positively associated with mortality risk, while housing stability, business density, and higher per capita income were protective. Suburban and rural municipalities showed the largest disparities in mortality risk, with distances to naloxone sites nearly eight times greater than in urban areas (IRR = 7.88, *p* = 0.003). Urban municipalities benefited from closer proximity to syringe access programs, which was associated with reduced mortality risk (IRR = 0.92, *p* = 0.011).

**Conclusion:**

Disparities in NMO mortality are shaped by SBEs that vary across urban, suburban, and rural municipalities. Housing instability, economic distress, and spatial access gaps in opioid health programs consistently contributed to elevated mortality, while stronger local economies and more stable housing were protective. These findings underscore that the risk of overdose mortality emerges through place-based conditions and call for strategies responsive to local SBEs, expanding affordable housing, strengthening community economies, and improving spatial access to harm reduction and treatment services across diverse geographic settings, as demonstrated in New Jersey.

**Supplementary Information:**

The online version contains supplementary material available at 10.1186/s12954-025-01332-7.

## Introduction

The opioid epidemic has revealed marked geographic disparities and shifts in mortality across the United States. Nonmedical opioid (NMO)^1^ use represents a substantial share of this burden, with opioids now accounting for nearly seven in ten overdose fatalities nationwide [1]. While opioid-related mortality remains persistent in major urban centers, over time it has also emerged as a major public health concern in rural and suburban areas, where opioid-involved deaths escalated between 1999 and 2015 (+ 325% in rural areas vs. + 198% in urban areas) [2]. By 2020, however, urban overdose death rates (28.6 per 100,000) slightly exceeded rural rates (26.2 per 100,000) [3, 4], underscoring the complex and shifting geography of opioid mortality risk. Some of the geographic shifts in overdose mortality are driven by changes in drug use patterns over time. Jenkins (2021) frames this evolving trajectory as four overlapping waves, prescription opioids, heroin, synthetic opioids, and a more recent stimulant/opioid wave, while noting that synthetic opioids have become the leading cause of overdose deaths in both urban and rural areas, heroin remains more prevalent in urban centers, and prescription opioids continue to drive mortality in rural areas [5–7].

These challenges are reflected in mortality patterns, as rural communities continue to experience disproportionately high deaths from prescription opioids and psychostimulants. Among women, for example, rural overdose death rates exceed those in urban areas (17.9 vs. 17.0 per 100,000) [3, 4]. Broader structural disadvantages intensify these risks, as rural areas often face economic distress, provider shortages, and limited behavioral health resources. Consequently, opioid-related mortality in rural communities has risen sharply, frequently outpacing rates in urban areas [4, 8–10]. These risks are not uniformly distributed; counties facing both economic hardship and healthcare shortages report especially high mortality [11].

Critical gaps in harm reduction services exacerbate overdose risk and contribute to adverse outcomes in rural communities. McLean (2016) documents limited naloxone access in deindustrialized rural areas [12], while Baker et al. (2024) report that rural people who use drugs often rely on unsafe overdose response strategies and avoid calling emergency services due to stigma and fear of law enforcement [13].

Taken together, these findings demonstrate how structural disadvantages and gaps in harm reduction services shape opioid-related risk and contribute to the geographically uneven burden of overdose mortality, reinforcing the importance of examining how place-based conditions, both social and built, drive differential NMO-related health outcomes.

Research on the built environment has largely focused on urban geography, where neighborhood conditions such as housing quality, disorder, infrastructure, and service accessibility have been shown to influence substance use, mental health, and overdose risk [14–22]. In contrast, predictors related to the built environment in suburban and rural areas remain underexamined, even though place-related conditions such as lower population density, limited transportation, and greater distances to healthcare and harm reduction services may shape NMO outcomes in distinct ways. To capture how features of the built environment influence opioid-related harm across diverse geographies, it is essential to broaden this research beyond urban settings.

Recognizing geographic differences is crucial for designing place-based interventions responsive to local risk environments. Identifying treatment and harm reduction “service deserts” can guide facility placement and support the expansion of telehealth and mobile health delivery. Geographic insights can also inform targeted resource allocation in low-resource areas and strengthen community initiatives addressing housing instability—an important determinant of NMO outcomes that both limits treatment access and increases the likelihood of high-risk use [23–25].

To examine these geographically driven dynamics, this study applies the Socio-Built Environment (SBE) framework [26], which integrates six domains spanning physical, residential, commercial, and social dimensions, along with community participation and spatial access to opioid-related services. Applying this multidimensional framework across urban, suburban, and rural contexts allows us to investigate how local place conditions contribute to disparities in NMO mortality. This approach extends prior research—often limited to rural vulnerability or urban built environments—by incorporating suburban settings and situating the analysis at the municipal level.

New Jersey provides an ideal case for this analysis: its varied geography includes dense urban cores, economically diverse suburbs, and isolated rural areas, enabling meaningful comparisons across social and built environments within a single state context. Findings can inform targeted interventions within the state while offering broader insights into other regions facing heterogeneous geographies and persistent opioid-related disparities.

### Geographic variation in NMO mortality: why urban, suburban, and rural contexts matter

Where individuals live—urban, suburban, or rural—shapes their risk of NMO mortality through differences in service availability, housing, and social conditions. Urban areas, characterized by high population density, typically offer a wide range of harm reduction services, including syringe access programs (SAPs), supervised injection sites, and mental health facilities that support individuals with opioid use disorder (OUD) [27]. The concentration of infrastructure and targeted funding in densely populated settings often makes these services more accessible [28]. Urban housing systems also more frequently include shelters, transitional housing, and supportive housing designed for people affected by substance use, reflecting a response to the needs of dense populations [29–31]. This clustering of city-based services and housing can help mitigate risks of NMO mortality.

Suburban and rural communities, by contrast, face distinct conditions that heighten risk. Geographic isolation and transportation barriers make treatment harder to access and sustain, while also increasing social isolation [32–34]. The absence of walkable neighborhoods and community spaces further weakens social support networks, reducing opportunities for harm reduction and recovery services [8, 21, 35]. Housing also plays a critical role: unlike cities, suburban and rural areas often lack supportive housing options, leaving individuals with substance use challenges more vulnerable to dependence and making recovery harder to maintain [23–25].

These geographic differences shape distinct pathways to disparities in NMO mortality and call for tools that can account for place-based risk environments. The SBE framework offers a way to identify these disparities and guide locally informed interventions. Applying this framework across urban, suburban, and rural settings highlights how risk is structured differently by place, and why effective strategies depend on understanding how risk is shaped by geography.

### Theoretically informed socio-built environment framework

The theoretical foundation of the SBE framework situates health within a socio-ecological system, emphasizing how social, economic, and political structures shape local conditions that influence well-being [36–38]. Disadvantaged communities often contend with limited resources, weak infrastructure, fewer opportunities for social interaction, and unsafe or poor-quality environments, all of which constrain individual choices and negatively affect mental health. In contrast, access to educational programs, job training, and supportive neighborhood resources can strengthen livelihoods and promote resilience [39]. In highly distressed communities, disadvantages are compounded by chronic stress and long-term disinvestment, conditions that further heighten susceptibility to OUD and increase the risk of NMO mortality [26]. Our theoretically driven SBE framework is outlined in Table 1.

**Table 1:** Socio-built environment domains and conceptual definitions informed by the literature

Built environment domains 1) *Quality of physical environment* (PhysEnv). This domain pertains to zoning and green environments of good quality (i.e., pleasant, agreeable)—including accessible green spaces, landscape aesthetics, and safe, walkable spaces. PhysEnv is linked to better health outcomes, including improved mental health and increased life expectancy [40–45] 2) *Quality of residential environment* (ResidEnv*).* The quality of the residential environment emphasizes having access to secure, affordable, and supportive housing. Such housing can elevate the quality of life and improve neighborhood settings, thereby fostering the health and well-being of vulnerable communities [17, 46–52] 3) *Quality of commercial environment* (ComEnv*)*. This domain considers the degree of local commercial quality environment concerning perceived safety and well-being. It measures vacant storefronts, abandoned buildings and foreclosures, and a lack of available public transportation. Localities with reduced (e.g., degradation) may face isolation, community disconnect, and service access barriers [52–59]	
Social Environment Domains 4) *Strength of community participation and social interaction* (CommPar). This component focuses on social capital, with particular reference to the role of volunteering in promoting social inclusion, assisting marginalized groups, and its relationship to other forms of civic participation and unpaid work [60–70]. Community participation also promotes and encourages social action in community building and renewal [70–72] 5) *Strength of community economic engagement* (CommEcon). *A strong* local economy and investments provide job opportunities and support the improvement of local infrastructure and community health and safety. CommEcon is fundamentally linked to indicators of quality of life, health, and overall well-being [49, 73]. As such, the fiscal health of a community is linked to sociability and residential isolation within a neighborhood, contributing to individuals’ stress and psychological well-being [52, 74–77] 6) *Spatial access to local opioid use-related health programs* (OHServ). Spatial access to opioid use health-related programs and harm reduction services plays a crucial role in helping to achieve and sustain treatment gains and optimize OUD health outcomes. [78–85]. Spatial access to local programs affects people’s ability to obtain services to reduce NMO-related risk when and where needed	

Empirical studies demonstrate that built environments are strong predictors of drug use, overdose mortality, and mental health outcomes in urban settings [16–19]. The strong link between the built environment, drug use, and mental health, underscores the importance of assessing built environment conditions in relation to OUD health outcomes and disparities, as these are directly connected to NMO mortality risk.

The social environment, in turn, encompasses the features and dynamics of social and cultural interactions among individuals within a community [61–64]. It includes interpersonal relationships, cultural norms, and patterns of civic engagement that shape levels of social support and collective efficacy [65–71]. Understanding these dynamics is important for assessing how community-level cohesion, participation, and resources influence vulnerability to OUD and the risk of NMO mortality.

Building on this foundation, our study advances research on opioid-related mortality risk environments by analyzing a comprehensive set of indicators across the six SBE domains in urban, suburban, and rural municipalities in New Jersey. In doing so, we provide a novel approach to understanding how geographic and socio-built environments shape disparities in NMO mortality. Brief definitions and descriptive statistics for these indicators are presented in Table 2 (Built Environment) and Table 3 (Social Environment), with detailed definitions and data sources available in Supplemental Table S2.

**Table 2:**
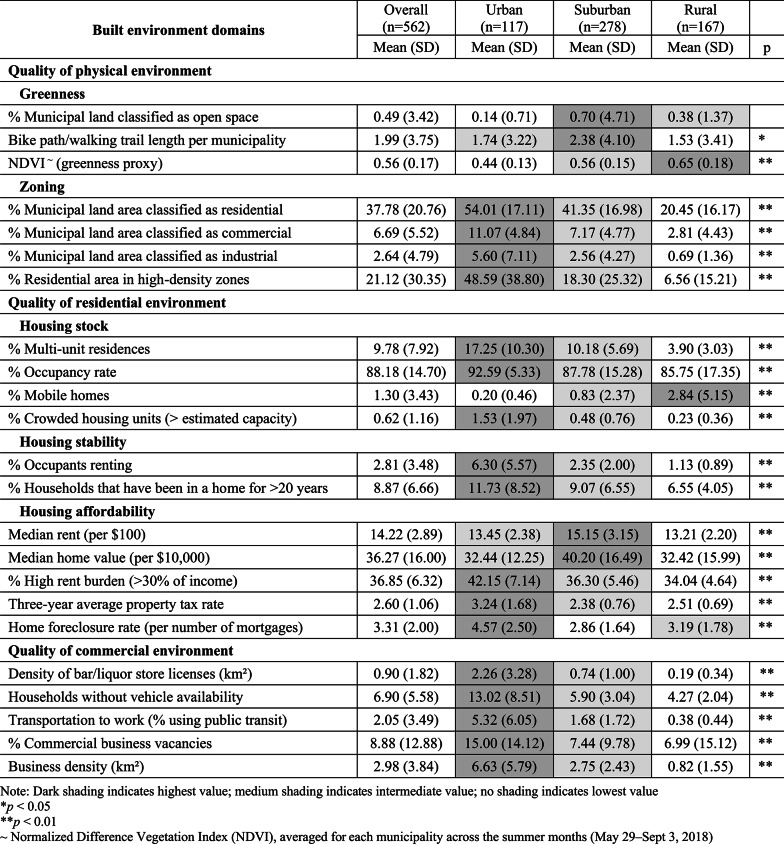
Built environment domains and indicators by urban, suburban, and rural municipalities, 2015–2018

**Table 3:**
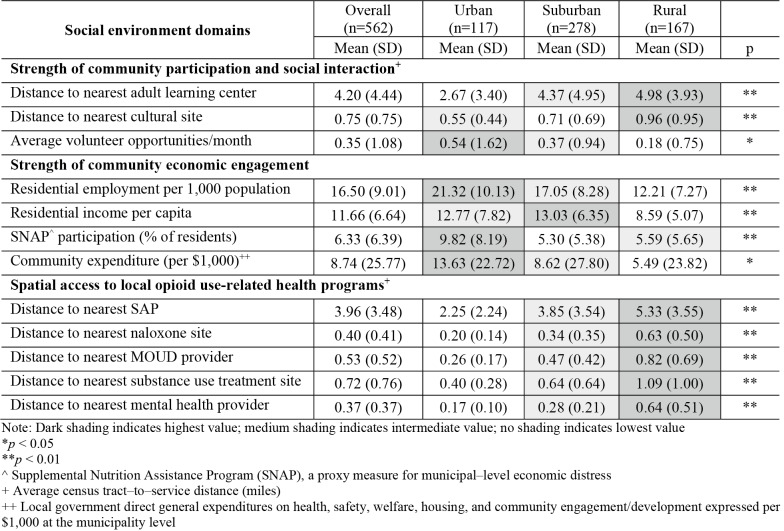
Social environment domains and indicators by urban, suburban, and rural municipalities, 2015–2018

## Methods

### Geographic sample

The geographic scale of analysis is the New Jersey (NJ) municipality level. Municipalities are legally defined local governments with authority over specific geographic areas, and together they cover the entire state of NJ. They reflect local population characteristics, economic conditions, and policy contexts, and are important for investigating NMO mortality because municipalities influence not only OUD treatment and harm reduction policies, but also local housing, zoning, and economic policies that shape community conditions. For this analysis, we collected data from 562 NJ municipalities: 117 urban, 278 suburban, and 167 rural. We used a method described by Williams et al. (2023) [86] to categorize each municipality as urban, suburban, or rural. This classification is based on the Degree of Urbanization indicator (DEGURB) [87–89], population density thresholds from the 2010 U.S. Census [90], and Rural–Urban Commuting Area (RUCA) Codes [91]. This classification allows us to assess how associations between the six SBE domains and NMO mortality vary across urban, suburban, and rural municipalities.

### Outcome measure

We used four years of drug overdose mortality data from the NJ Office of the Chief State Medical Examiner [92, 93] to identify NMO-related deaths, 2015–2018. For deaths identified as opioid-related, data were extracted and georeferenced based on residential addresses. Deaths with incomplete address information were omitted from the dataset. The final dataset comprises 7,496 observations, representing approximately 80% of the original 9,324 opioid-related deaths. Out of the 7,496 NMO deaths reported, 68% occurred at the place of residence.

Next, we implement a spatial join process in R v3.22 to consolidate geocoded NMO-related deaths within municipal boundaries. To address the issue of small annual counts of NMO-related deaths within municipalities, we use as the outcome variable the total opioid-related deaths over four years, 2015–2018. To measure population base, we use data from the American Community Survey’s 2017 5-year population estimates by municipality [94]. We conduct a cross-sectional analysis to determine associations between the risk of NMO overdose mortality and SBE indicators of the environment across urban, suburban, and rural geographies. Lastly, we include municipality-level gender, race/ethnicity, and education demographic characteristics as covariates in the model to address potential confounding effects.

### Measures of socio-built environments

We operationalized the six domains of the SBE framework [26] using indicators drawn from secondary data sources. Built environment measures included land use composition (residential, commercial, industrial), greenness (walking paths, bike trails, Normalized Difference Vegetation Index (NDVI)), housing stock and stability (multi-unit structures, occupancy, rental rates, residential tenure), and commercial activity. Social environment measures included community participation (distance to adult learning and cultural centers, volunteer opportunities), community economic engagement, and spatial access to opioid-related health services (distance to treatment programs, syringe access programs, and other harm reduction services). Summary statistics are presented in Table 2 (Built Environment) and Table 3 (Social Environment), with detailed definitions and data sources available in Supplemental Table S2.

### Analytical approach

#### Model

Due to the highly skewed and over-dispersed nature of the NMO mortality outcome variable, we fit a generalized linear model (GLM) with a negative binomial outcome distribution, incorporating the log of the population as an offset to estimate the effect of SBE on NMO mortality risk (96–98] and estimate from the GLM the incidence rate ratio (IRR) of NMO overdose mortality [98, 99]. The IRR quantifies the relationship between SBE and the likelihood of NMO overdose mortality; values greater than 1 suggest a positive association, while values less than 1 indicate an inverse relationship. An IRR of 1 reflects no association.

#### Analysis

We compared mean SBE indicator values across urban, suburban, and rural municipalities using one-way ANOVA. Next, to analyze the association between each SBE domain and NMO overdose mortality, we fit a separate GLM for each of the six domains, adjusting for demographic factors (age distribution, race/ethnicity, and sex composition). Within each domain model, all indicators from that domain were entered simultaneously as predictors. Additionally, we evaluated the overall influence of all indicators within each domain on NMO overdose mortality by comparing the log-likelihood statistics between models with and without the target domain variables. We performed the log-likelihood ratio test to evaluate the statistical significance of the change in model fit. A significant test result suggests that the domain as a whole has a measurable impact on the risk of NMO overdose mortality. Because the SBE framework conceptualizes domains as multidimensional constructs, we report both the domain-level effect (via likelihood ratio *χ*2 tests) and the specific effects of individual indicators (via IRRs). This dual approach highlights the overall influence of each domain while retaining interpretability of individual associations.

Models were estimated first for the full sample and then separately for urban, suburban, and rural municipalities, enabling comparisons of IRR values across geographic contexts. The number of municipalities in the sample (n = 562) exceeded the number of predictor variables in each model by a ratio well above the commonly recommended 10:1 threshold, minimizing the risk of overfitting [100]. Multicollinearity was assessed using Variance Inflation Factor (VIF) values for all predictors within each domain. Statistical significance was evaluated at the 5% level, and all analyses were conducted using SAS software, version 9.4 [101].

## Results

### Comparative patterns of socio-built environment indicators across urban, suburban, and rural geographies^2^

Table 2 presents average values for built environment indicators across urban, suburban, and rural municipalities in New Jersey. In the PhysEnv domain, urban municipalities show substantially higher averages for all land use indicators—including the proportion of land designated for residential, commercial, and industrial use—as well as residential structure density. When it comes to greenness, suburban areas have the greatest lengths of walking paths and bike trails, while rural municipalities report the highest NDVI, reflecting greater vegetative cover. In the ResidEnv, urban municipalities report the highest average values for greater economic distress housing stock—specifically, the proportion of multi-unit residences, occupancy rates, and crowded housing units—as well as housing stability, with higher percentages of renters and households residing in the same home for over 20 years. Finally, all measures within the ComEnv domain, such as business density, liquor store licensing, public transit use, and commercial vacancy, are highest in urban municipalities, demonstrating the impact of the built environment in these areas.

Table 3 displays the average values of social environment indicators across urban, suburban, and rural municipalities. In the CommPar domain, rural municipalities show the greatest average distances to adult learning centers and cultural points of interest, reflecting lower accessibility to these resources. Urban municipalities, in contrast, report the highest average number of volunteer opportunities, suggesting a denser network of civic engagement. In the CommEcon domain, urban municipalities record the highest mean values across all indicators, including residential employment per 1,000 residents, local government spending, and SNAP participation rates. Finally, and most notably, in the domain of OHServ, rural municipalities consistently report the longest average distances to all service types, including syringe access programs, naloxone sites, MOUD providers, treatment centers, and mental health services, indicating substantial disparities in access compared to urban and suburban areas.

### Geographic variation in socio-built environment domain-level associations with NMO mortality risk

As described in the Methods, each domain-specific GLM simultaneously included all indicators from the domain and adjusted for demographic covariates to account for potential confounding. Across all domain-wise models, the sample-to-predictor variable ratio was greater than 10:1, indicating a low risk of overfitting. Variance Inflation Factor (VIF) values were also examined to assess multicollinearity. While a few predictors had VIF values greater than 5, all remained below the commonly accepted threshold of 10, suggesting no evidence of serious multicollinearity [102].

**Figure 1:**
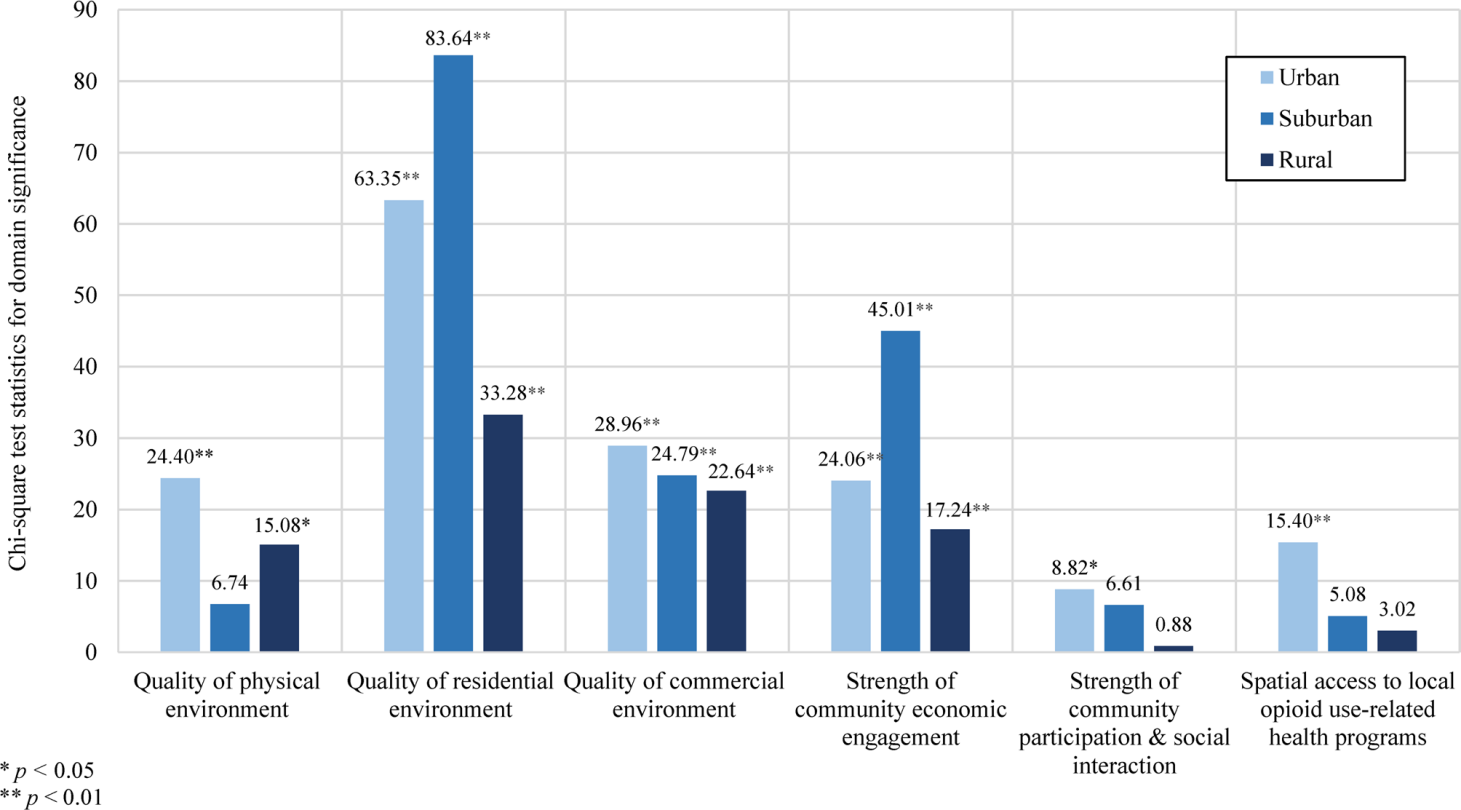
Domain-level contributions of socio-built environments to NMO mortality risk across urban, suburban, and rural geographies

Figure 1 displays *χ*^2^ values from likelihood ratio tests, summarizing the domain-level effects of each SBE construct across urban, suburban, and rural municipalities. This provides an overview of which domains exert the strongest influence on NMO mortality risk, complementing the indicator-level results shown in Table 4. In contrast, Table 4 reports indicator-level associations from each domain-specific GLM, where all indicators in the domain were modeled together while controlling for demographic covariates. Results are shown as IRRs, 95% confidence intervals (CIs), and p-values for individual indicators, along with likelihood ratio *χ*^2^ statistics assessing the joint contribution of each domain.

**Table 4:**
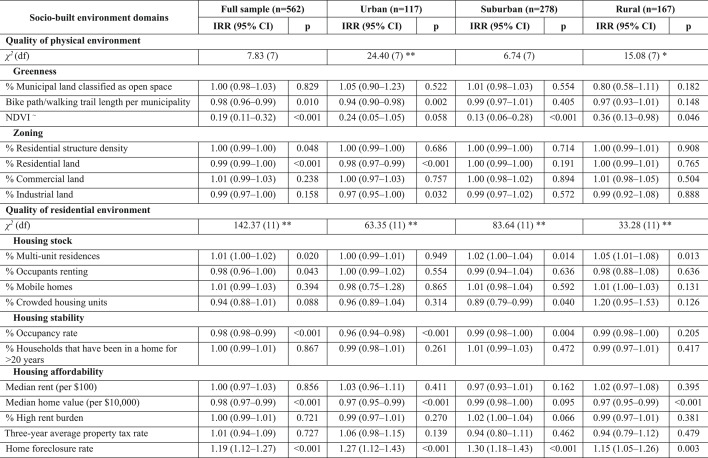

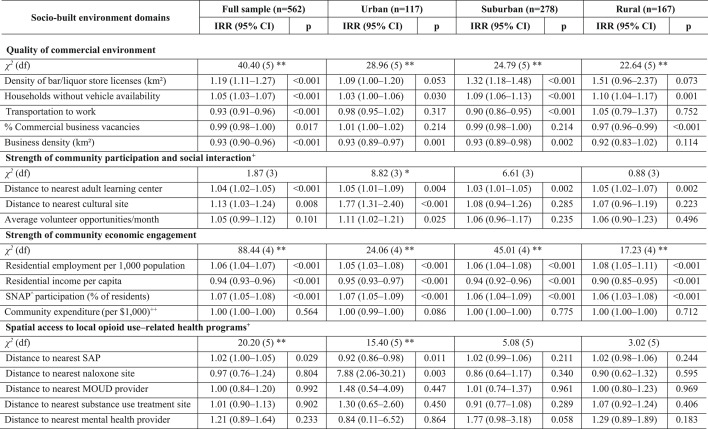

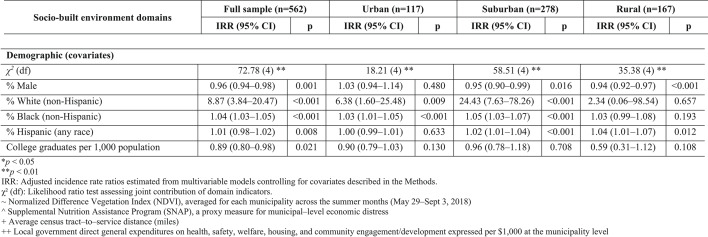
Indicator-level associations of socio-built environment domains with NMO mortality risk across geographies

#### Quality of physical environment

The PhysEnv domain was significantly associated with NMO mortality in urban (*χ*^2^ = 24.40, *p* < 0.01) and rural municipalities (*χ*^2^ = 15.08, *p* < 0.05), but not in suburban areas. At the indicator level, higher greenness (NDVI) was inversely associated with mortality across the full sample and in suburban and rural municipalities, while greater length of walking paths and bike trails was protective in urban areas.

#### Quality of residential environment

The ResidEnv domain was strongly associated with mortality across all geographies (full sample *χ*^2^ = 142.37, urban *χ*^2^ = 63.35, suburban *χ*^2^ = 83.64, rural *χ*^2^ = 33.28; all *p* < 0.01). Key indicators included housing instability, with foreclosure rates consistently linked to higher mortality across geographies. In suburban and rural areas, greater shares of multi-unit housing were also associated with increased risk, while higher occupancy rates were protective in urban and suburban municipalities.

#### Quality of commercial environment

Within the ComEnv domain, the full-sample model (*χ*^2^ = 40.40, *p* < 0.01) revealed significant associations with NMO mortality. These associations held across urban (*χ*^2^ = 28.96, *p* < 0.01), suburban (*χ*^2^ = 24.79, *p* < 0.01), and rural municipalities (*χ*^2^ = 22.64, *p* < 0.01). At the indicator level, higher density of bar and liquor store licenses and greater proportions of households without vehicle access were positively associated with NMO mortality risk, particularly in suburban and rural areas. By contrast, greater business density and higher public transit use were inversely associated with mortality in the full sample and suburban municipalities.

#### Strength of community economic engagement

Community economic engagement was significantly associated with mortality across all geographies (full sample *χ*^2^ = 88.44, urban *χ*^2^ = 24.06, suburban *χ*^2^ = 45.01, rural *χ*^2^ = 17.23; all *p* < 0.01). Higher per capita income was protective, while residential employment showed a positive association with mortality across geographies. In addition, higher municipal SNAP rates were positively associated with mortality risk in every geographic setting.

#### Strength of community participation and social interaction

The CommPar domain, which represents access to community resources and social capital, reached significance only in urban municipalities (*χ*^2^ = 8.82, *p* < 0.05). Distance to adult learning centers and cultural institutions was positively associated with mortality in the full sample and in urban municipalities, while availability of volunteer opportunities showed a modest protective association in urban areas.

#### Spatial access to local opioid use-related health programs

Spatial access to opioid-related health programs was statistically associated with mortality risk in both the full-sample model (*χ*^2^ = 20.20, *p* < 0.01) and in urban municipalities (*χ*^2^ = 15.40, *p* < 0.01). Among individual indicators, distance to SAPs was positively associated with mortality risk in the full sample but inversely associated in urban municipalities. However, as Fig. 1 shows, the domain’s *χ*^2^ values are modest compared to other SBE domains, and its contribution is weaker in suburban and rural municipalities, where associations were not significant. This suggests that while program access plays a role in urban settings, its effect is less consistent across other geographies.

### Geographic variation in NMO mortality disparities across socio-built environment domains

Three domains demonstrated consistent and statistically significant associations with NMO overdose mortality across the full sample and all geographic areas. Across NJ municipalities, variation in commercial, residential, and economic environments was consistently associated with disparities in mortality risk.

#### Quality of commercial environments

The commercial environment emerged as a statistically significant predictor of NMO mortality across all geographies, with associations holding in the full-sample, urban, suburban, and rural models (*p* < 0.01). At the indicator level, higher densities of bar and liquor store licenses and greater proportions of households without vehicle access were positively associated with NMO mortality, particularly in suburban and rural municipalities. Conversely, greater business density and public transit use were associated with reduced mortality risk, especially in suburban areas.

#### Quality of residential environments

Housing instability, captured by foreclosure rates, rent burdens, and broader affordability pressures, was positively associated with NMO mortality across municipalities. In contrast, municipalities with more stable and affordable housing patterns, including higher occupancy rates, showed lower risk.

#### Strength of community economic engagement

Higher per capita income was protective across all models. In contrast, residential employment showed a positive association with mortality risk across geographies, and higher municipal SNAP rates were consistently associated with greater mortality risk. Across urban, suburban, and rural municipalities, each percentage-point increase in municipal SNAP rates corresponded to an estimated 5–7% increase in mortality risk. Community investment was not significantly associated with mortality.

## Discussion

### Summary of findings

This study examined how geographic and socio-built environmental conditions contribute to disparities in NMO mortality across New Jersey municipalities. Using the SBE framework, we found that NMO mortality is consistently shaped by place-based conditions that vary across urban, suburban, and rural geographies.

Our domain-level analysis underscores the multidimensional influence of socio-built environments, while indicator-level estimates offer more granular insight into specific mechanisms of risk, such as liquor outlet density, foreclosure rates, and SNAP enrollment. These findings illustrate the value of analyzing SBEs both as integrated domains and as distinct, actionable indicators. As shown in Fig. 1, the relative influence of each domain varies across geographies, reinforcing the importance of geographic-specific approaches to understanding and addressing NMO mortality.

The built environment emerged as especially significant in suburban and rural municipalities, where housing instability, limited healthcare access, and economic disinvestment heighten vulnerability to overdose. Structural challenges, such as high rent burdens in suburban areas and limited housing stock and service access in rural ones, underscore the importance of improving housing affordability, expanding transportation, and strengthening local economies. In these contexts, mobile health units and telehealth platforms may help close service gaps where conventional models fall short, while investments in stable, affordable housing may mitigate upstream drivers of NMO-related harm.

The commercial environment emerged as the most consistent predictor of NMO mortality risk, driven by factors such as liquor outlet density, vacant storefronts, and broader patterns of economic disinvestment. In both suburban and rural municipalities, these indicators were strongly associated with elevated mortality, extending prior research on alcohol outlet density and overdose [55, 103–107]. These findings reinforce the conceptual foundation of the ComEnv domain, which captures neighborhood-level markers of distress, including abandoned buildings, commercial vacancy, and limited public transit, as conditions that can erode perceived safety, disrupt social cohesion, and restrict access to services [52–59]. In this context, decline in the commercial environment contributes not only to increased risk but also to disparities in NMO across municipalities. Limited transportation access further compounds this vulnerability, aligning with evidence that infrastructure gaps shape health outcomes, including overdose [105, 107]. The geographic patterns observed, particularly in suburban and rural areas, underscore how zoning and development policy can either reinforce or help remediate the commercial conditions that shape NMO mortality risk. Targeted planning reforms that reduce NMO-related harm and promote infrastructure investment may help mitigate NMO disparities and advance place-based health equity in the most affected communities.

The residential environment domain was a strong and consistent predictor of NMO mortality across all geographies, reinforcing the theoretical premise that access to secure, affordable, and stable housing is foundational to health and well-being. High foreclosure rates were associated with increased mortality in urban, suburban, and rural municipalities, highlighting the role of housing instability as a structural driver of overdose risk. Foreclosures reflect economic distress, displacement, and the breakdown of neighborhood stability, conditions that can erode protective social networks and increase vulnerability to NMO-related harms [23–25, 48, 73, 77]. In suburban and rural areas, greater shares of multi-unit housing were also linked to elevated mortality, which may signal the presence of lower-quality or less stable housing stock in areas where rental options are limited or stigmatized. Conversely, higher occupancy rates were protective in urban and suburban settings, suggesting that neighborhood stability and reduced housing turnover may buffer against risk of NMO mortality. These findings support the theoretical framing of our residential environment domain as a critical determinant of place-based NMO-related outcomes and underscore the importance of housing policy in mitigating geographic disparities in NMO mortality and advancing harm reduction policies related to safe and affordable housing [23–25, 48, 77].

Economic vulnerability was a consistent predictor of NMO mortality. Municipalities with stronger local economies and higher per capita incomes had lower mortality rates, reflecting the protective effects of economic stability [108]. However, employment itself was not uniformly protective. In our models, higher levels of residential employment were associated with increased mortality, which may reflect local labor markets shaped by high-risk, low-wage, or unstable work environments that sustain vulnerability rather than mitigate it [109]. In contrast, municipalities with higher levels of economic distress, as proxied by SNAP participation rates, experienced a 5–7% increase in NMO mortality risk for each percentage-point increase in SNAP participation. This pattern likely reflects broader structural inequities and community-level health burdens in economically distressed municipalities, rather than any direct effect of SNAP participation [110–113]. Our community economic engagement domain was developed to capture the financial health of municipalities and the fiscal conditions that shape pathways influencing neighborhood quality, community well-being, and local harm. While broader redistributive policies, such as progressive taxation and housing subsidies, were not directly examined, our findings support their relevance for future research.

Lastly, suburban and rural municipalities in New Jersey face some of the greatest challenges in accessing OUD-related services, contributing to elevated risk for NMO mortality. The average distance to harm reduction and treatment services, such as SAPs, naloxone sites, and MOUD providers, is substantially greater in these areas compared to urban municipalities, underscoring a pronounced disparity in service availability. In both the full-sample and urban models, greater distance to OUD-related services was significantly associated with higher NMO mortality risk; however, this relationship was not observed in the suburban and rural models, reflecting weaker or inconsistent spatial access effects outside urban settings. Two contextual factors make help shape this pattern. First, harm reduction infrastructure is heavily concentrated in urban centers, where population density and service integration support program viability, leaving less-populated areas with limited geographic coverage [114, 115]. Second, efforts to expand SAPs and related services into higher-resource or residential communities often encounter not-in-my-backyard (NIMBY) resistance, limiting their distribution beyond urban cores [83, 116, 117]. These findings highlight not only geographic gaps in harm reduction services but also the ways in which local political and social dynamics influence whether vulnerable communities can access lifesaving OUD–NMO-related supports.

### The geography of risk: implications for harm reduction policy

Overall, these findings indicate that disparities in NMO mortality are rooted in spatially structured vulnerabilities across socio-built environments. Commercial disinvestment, housing instability, economic distress, and gaps in service access consistently emerged as risk factors, especially in suburban and rural municipalities, settings that remain underexamined in overdose research. By applying the SBE framework across multiple geographies, this study moves beyond prior urban-focused work and provides new evidence on how local place-based conditions shape NMO mortality in suburban and rural context.

SBE conditions also intersect in ways that reinforce geographic disparities. Housing instability is closely tied to economic inequity, compounding barriers to NMO treatment and care. In suburban areas, high housing costs and limited transit can push residents farther from treatment and harm reduction services. In rural municipalities, economic disinvestment and geographic isolation coincide with sparse healthcare infrastructure. In these contexts, distance to care is not only geographic but also reflects structural inequities embedded in place-based housing and economically distressed environments.

Policy responses should account for these geographic differences. In suburban and rural areas, expanding affordable housing, strengthening local economies, and improving transit access remain essential. Mobile health units and telehealth may help close service gaps where traditional care models are less viable. In New Jersey, where municipalities range from dense urban centers to suburban and rural communities, tailoring interventions to local SBE conditions is critical to building effective and equitable responses to NMO mortality. This includes addressing treatment and harm reduction “service deserts” through targeted siting of facilities, mobile health delivery, and expanded telehealth to ensure access across suburban, rural, and other underserved communities.

### Limitations

This study has several limitations. First, overdose events were geocoded to the decedent’s residence rather than to the actual location of the overdose (e.g., motel, park, jail) due to restrictions in the NJOSME data. This limits our ability to capture possible discordance between where overdoses occur and residential exposures across SBE domains. However, prior research indicates that most overdoses occur in the home, suggesting that residential location remains a reasonable proxy for exposure [118, 119].

Second, approximately 20% of overdose cases lacked complete geocoded address information and were excluded from the analysis. We were unable to determine whether these missing cases were systematically associated with factors such as homelessness, housing instability, or urbanicity, and this may have introduced selection bias.

Third, the study focuses exclusively on fatal NMO overdoses. Non-fatal overdoses, which may be shaped by distinct sets of SBE risk factors, needs, and resources, were not captured in this analysis. In addition, the cross-sectional design precludes causal inference, and the associations observed here should be interpreted as correlational rather than causal.

Fourth, the SBE indicators were derived primarily from secondary data sources. As a result, we were limited in our ability to control data quality and consistency, and some potentially relevant indicators, such as direct measures of neighborhood conditions or area-level buprenorphine prescribing, were not available. Future research should build on this framework by incorporating more fine-grained measures, accounting for temporal change, and examining how shifts in SBE domains over time shape geographic variation in mortality.

Finally, this study covers the pre-COVID-19 period (2015–2018). Local conditions have changed substantially since then, with implications for housing stability, health service availability, and overdose risk patterns. While these shifts should be considered when interpreting our findings, the study nonetheless provides a valuable baseline for understanding pre-pandemic associations between place-based conditions and NMO-related risk and demonstrates the utility of the SBE framework for identifying geographically embedded disparities.

## Conclusion

This study demonstrates that the geography of risk in NMO mortality is shaped by socio-built environments that vary across urban, suburban, and rural municipalities in New Jersey. Housing instability, economic distress, and spatial access gaps in opioid use–related health services were consistently associated with elevated mortality risk, whereas stronger local economies and more stable housing conditions were protective. These findings indicate that risk of overdose mortality is not evenly distributed but arises from place-specific social and built conditions. Addressing these disparities will require locally tailored strategies that expand housing stability, strengthen community economies, and improve access to harm reduction and treatment services. Although this analysis is grounded in New Jersey, its insights advance understanding of the geography of risk and remain relevant for other regions characterized by diverse geographies and persistent opioid-related disparities.

## Supplementary Information

Supplementary material 1

Supplementary material 2

## Data Availability

The data used in this study are not publicly available due to privacy and confidentiality concerns related to sensitive health and mortality records. Access to the data is restricted to protect the privacy of individuals and communities included in the analysis. Researchers interested in access may contact the corresponding author for information about data sources and potential data use agreements, subject to institutional and ethical approval.

## Abbreviations

NMO: Nonmedical opioid
SBE: Socio-built environments
PhysEnv: Quality of physical environment
ResidEnv: Quality of residential environment
ComEnv: Quality of commercial environment
CommPar: Strength of community participation and social interaction
CommEcon: Strength of community economic engagement
OHServ: Spatial access to local opioid use-related health programs
NDVI: Normalized difference vegetation index
MOUD: Medications for opioid use disorder
OUD: Opioid use disorder
SAP: Syringe access program
SNAP: Supplemental nutrition assistance program
MAT: Medication-assisted treatment
GLM: Generalized linear model
IRR: Incidence rate ratio
CI: 95% Confidence interval
VIF: Variance inflation factor
NJ: New Jersey
NJOCSME: NJ office of the Chief state medical examiner
NIMBY: Not-in-my-back-yard

## Footnotes

1 Nonmedical opioids (NMO), as defined in this study, include natural and semi-synthetic opioids, synthetic opioids, and heroin, encompassing both the non-prescribed use of pharmaceutical opioids and the use of illicit opioids.

2 Descriptive statistics for NJ municipality-level population distribution can be found in Supplemental material Table S1.
