# Supplementary material for: The geography of risk: understanding disparities in nonmedical opioid mortality and the role of socio-built environments in New Jersey
Source: Harm Reduct J. 2026 Feb 21;23:124. doi: 10.1186/s12954-025-01332-7 (PMC13393885; doi:10.1186/s12954-025-01332-7)
Supplement: Supplementary file 1 — Supplementary material 1 [file 12954_2025_1332_MOESM1_ESM.docx]

| **Table S1.** Municipality-Level Descriptive Statistics by Urban, Suburban, and Rural Geographies, New Jersey (2015–2018) | | | | | |
| --- | --- | --- | --- | --- | --- |
|  | Overall  (n=562) | Urban  (n=117) | Suburban  (n=278) | Rural  (n=167) |  |
|  | Mean (SD) | Mean (SD) | Mean (SD) | Mean (SD) |  |
|  |  |  |  |  |  |
| Opioid death per 10k population | 13.7 (13.7) | 12.5 (11.9) | 14.0 (14.9) | 13.3 (13.0) |  |
| **Gender** | | | | | |
| % Male | 48.9 (3.1) | 48.6 (1.8) | 48.4 (2.1) | 49.9 (4.7) | ** |
| % Female | 51.2 (3.6) | 51.4 (1.8) | 51.6 (2.1) | 50.3 (5.7) | ** |
| **Racial/Ethnicity** | | | | | |
| % White | 79.9 (17.4) | 65.5 (21.0) | 80.9 (14.4) | 88.2 (12.2) | ** |
| % Black | 7.7 (11.8) | 13.4 (16.9) | 6.7 (10.0) | 5.5 (8.8) | ** |
| % Hispanic | 12.5 (13.3) | 24.4 (19.4) | 10.9 (9.9) | 7.1 (5.9) | ** |
| **Education** |  |  |  |  |  |
| College graduate per 1,000 population | 3.7 (5.5) | 6.2 (8.6) | 4.1 (4.4) | 1.4 (1.6) | ** |
| *** p<.001* Comparing Geographies  Data source: New Jersey State Data Center, 2022 | | | | | |
