# Supplementary material for: The geography of risk: understanding disparities in nonmedical opioid mortality and the role of socio-built environments in New Jersey
Source: Harm Reduct J. 2026 Feb 21;23:124. doi: 10.1186/s12954-025-01332-7 (PMC13393885; doi:10.1186/s12954-025-01332-7)
Supplement: Supplementary file 2 — Supplementary material 2 [file 12954_2025_1332_MOESM2_ESM.docx]

| **Table S2.** Municipality-Level Indicators and Data Sources for the Socio-Built Environment Framework | | | | | |
| --- | --- | --- | --- | --- | --- |
| **Domain** | **Sub Category** | **Description** | **Year(s)** | **Source** | **Citation** |
| Quality of Physical Environment | Greenness | Percent of all land that is state/local/nonprofit open land, as a proxy for proportion of parks | 2022 | New Jersey Geographic Information Network (NJGIN) Open Data portal | NJ Office of GIS. (2022). New Jersey Geographic Information Network Open Data. <https://njogis-newjersey.opendata.arcgis.com>. Accessed April 1, 2022. |
|  |  | Sum of lengths of all bike trails and pedestrian paths | 2020 | OpenStreetMap | OpenStreetMap contributors. (2020). Planet dump [Data file from 2020.10.01]. Retrieved from <https://planet.openstreetmap.org>. Accessed November 2, 2020. |
|  |  | Average monthly Normalized Difference Vegetation Index (NDVI) over the course of the summer, as a proxy for green space | 2018 (May-Sept.) | U.S. Geological Survey, EarthExplorer | USGS. (2020). EarthExplorer. 2018 Normalized Difference Vegetation Index Data. <https://earthexplorer.usgs.gov/>. Accessed February 13, 2020. |
|  | Zoning | Proportion of residential area that is high density | 2015 | Rowan University Geodata Center, New Jersey MAP | RUGC. (2020). New Jersey MAP: Land Use Change 1986-2015. <https://www.njmap2.com/landuse/landuse/>. Accessed November 2, 2020. |
|  |  | Proportion of all municipal land that is residential | 2015 | Rowan University Geodata Center, New Jersey MAP | RUGC. (2020). New Jersey MAP: Land Use Change 1986-2015. <https://www.njmap2.com/landuse/landuse/>. Accessed November 2, 2020. |
|  |  | Proportion of all municipal land that is commercial | 2015 | Rowan University Geodata Center, New Jersey MAP | RUGC. (2020). New Jersey MAP: Land Use Change 1986-2015. <https://www.njmap2.com/landuse/landuse/>. Accessed November 2, 2020. |
|  |  | Proportion of all municipal land that is industrial | 2015 | Rowan University Geodata Center, New Jersey MAP | RUGC. (2020). New Jersey MAP: Land Use Change 1986-2015. <https://www.njmap2.com/landuse/landuse/>. Accessed November 2, 2020. |
| Quality of Commercial Environment |  | Number of liquor licenses given to pubs/bars/shops per kilometer squared | 2019 | New Jersey Office of Attorney General (NJOAG) | NJOAG. (2021). Division of Alcoholic Beverage Control. Alcoholic Beverage Permits & Licenses. [www.njoag.gov](file:///C:\Users\LWilliams\Dropbox\BE&OM\www.njoag.gov). Accessed Sep 8, 2021. |
|  |  | Households without vehicle availability | 2018 five-year estimate | United States Census Bureau, American Community Survey | USCB. (2021). American Community Survey. <https://www.census.gov/data.html>. Accessed November 9, 2020. |
|  |  | Percentage of people commuting to work via public transit | 2018 five-year estimate | United States Census Bureau, American Community Survey | USCB. (2021). American Community Survey. <https://www.census.gov/data.html>. Accessed November 29, 2020. |
|  |  | Percentage of vacant businesses | 2018 | United States Department of Housing and Urban Development (HUD) | HUD. (2020). Office of Policy Development and Research. Aggregated United States Postal Service Administrative Data. <https://www.huduser.gov/portal/datasets/usps.html> Accessed October 20, 2020. |
|  |  | Number of businesses per kilometer squared | 2019 | InfoGroup, Inc. | InfoGroup. (2019). Historical Business Files, New Jersey. <https://dataverse.harvard.edu/dataset.xhtml?persistentId=doi:10.7910/DVN/PNOFKI>. Accessed November 18, 20, 2020. |
| Quality of Residential Environment | Housing Stock | Mobile home percentage | 2018 five-year estimate | United States Census Bureau, American Community Survey | USCB. (2021). American Community Survey. <https://www.census.gov/data.html>. Accessed November 9, 2020. |
|  |  | Multi-unit residences percentage | 2018 five-year estimate | United States Census Bureau, American Community Survey | USCB. (2021). American Community Survey. <https://www.census.gov/data.html>. Accessed November 9, 2020. |
|  |  | Crowded housing unit percentage (estimate of number of households with more people than rooms) | 2018 five-year estimate | United States Census Bureau, American Community Survey | USCB. (2021). American Community Survey. <https://www.census.gov/data.html>. Accessed November 9, 2020. |
|  |  | Percent of occupants renting | 2018 five-year estimate | United States Census Bureau, American Community Survey | USCB. (2021). American Community Survey. <https://www.census.gov/data.html>. Accessed November 9, 2020. |
|  | Housing Stability | Occupancy rate | 2018 five-year estimate | United States Census Bureau, American Community Survey | USCB. (2021). American Community Survey. <https://www.census.gov/data.html>. Accessed November 9, 2020. |
|  |  | Percent of households that have been there for more than twenty years | 2018 five-year estimate | United States Census Bureau, American Community Survey | USCB. (2021). American Community Survey. <https://www.census.gov/data.html>. Accessed November 9, 2020. |
|  | Affordability | Three-year effective property tax rate | 2017 | New Jersey Department of Community Affairs (NJDCA) | NJDCA. (2020). The 2017 Municipal Revitalization Index. [https://nj.gov/dca](https://nj.gov/dca/). Accessed December 9, 2020. |
|  |  | Median rent | 2020 | New Jersey Department of Community Affairs (NJDCA) | NJDCA. (2021). Neighborhood Revitalization Tax Credit Program (NRTC). <https://www.nj.gov/dca/divisions/dhcr/offices/nrtc.html>  Accessed August 10, 2021. |
|  |  | Median home value | 2020 | New Jersey Department of Community Affairs (NJDCA) | NJDCA. (2021). Neighborhood Revitalization Tax Credit Program (NRTC). <https://www.nj.gov/dca/divisions/dhcr/offices/nrtc.html>  Accessed August 10, 2021. |
|  |  | Percent of households with a ratio of housing costs to income in excess of 30% | 2020 | New Jersey Department of Community Affairs (NJDCA) | NJDCA. (2021). Neighborhood Revitalization Tax Credit Program (NRTC). <https://www.nj.gov/dca/divisions/dhcr/offices/nrtc.html>  Accessed August 10, 2021. |
|  |  | Foreclosure rate (per number of mortgages) | 2010 | United States Department of Housing and Urban Development (HUD) | HUD. (2010). Office of Policy Development and Research. Neighborhood Stabilization Program Data. <https://www.huduser.gov/portal/datasets/NSP.html>. Accessed August 13, 2021. |
| Local Opioid Use Related Programs/Services |  | Average distance from each Census tract to the nearest naloxone-carrying pharmacy (miles) | 2020 | New Jersey Department of Human Services (NJDHS) | NJDHS (2021). Naloxone Distribution at Participating Pharmacies. <https://www.nj.gov/humanservices/stopoverdoses/>. Accessed August 4, 2021. |
|  |  | Average distance from each Census tract to the nearest syringe access program (miles) | 2020 | New Jersey Department of Health (NJDOH) | NJDOH. (2021). Harm Reduction Centers. <https://www.nj.gov/health/hivstdtb/sap.shtml>.  Accessed August 4, 2021. |
|  |  | Average distance from each Census tract to providers prescribing medications for opioid overuse disorder (miles) | 2020 | Substance Abuse and Mental Health Services Administration (SAMHSA) | SAMHSA (2021). SAMHSA Treatment Finder.  <https://findtreatment.samhsa.gov/locator>. Accessed 7/20/2021. |
|  |  | Average distance from each Census tract to the nearest substance use treatment center (miles) | 2020 | Substance Abuse and Mental Health Services Administration (SAMHSA) | SAMHSA (2021). SAMHSA Treatment Finder.  <https://findtreatment.samhsa.gov/locator> Accessed 7/20/2021. |
|  |  | Average distance from each Census tract to nearest mental health provider (miles) | 2020 | Substance Abuse and Mental Health Services Administration (SAMHSA) | SAMHSA (2021). SAMHSA Treatment Finder.  <https://findtreatment.samhsa.gov/locator> Accessed 7/20/2021. |
| Strength of Community Participation and Social Interaction |  | Average distance from each Census tract to the nearest adult education program (miles) | 2020 | New Jersey Geographic Information Network (NJGIN) Open Data portal | NJ Office of GIS. (2021). New Jersey Geographic Information Network Open Data. School Point Locations of NJ (Public, Private, and Charter). <https://njogis-newjersey.opendata.arcgis.com/datasets/school-point-locations-of-nj-public-private-and-charter/explore?location=40.105701%2C-74.739700%2C8.99>. Accessed April 21, 2021. |
|  |  | Average distance from each Census tract to the nearest cultural points (cultural center, art gallery, or museum) (miles) | 2021 | Google Search for “Cultural centers,” “art galleries,” and “museums” in each municipality. | N/A. We collected addresses for each cultural center found and used them to compute the distance from each Census tract within the municipality to the nearest cultural center. Google search completed August 1, 2021. |
|  |  | Average number of volunteer opportunities per month | 2021 | <https://www.jerseycares.org/>  and  <https://www.volunteermatch.org/search?l=NJ> | N/A. Between February 2021 and May 2021, we conducted searches of volunteerism opportunities on both websites, at least once a month, and computed the average number of opportunities for each municipality. |
| Strength of Community Economic Engagement |  | Residential employment per 1,000 population | 2018 five-year estimate | United States Census Bureau, American Community Survey | USCB. (2021). American Community Survey. <https://www.census.gov/data.html>. Accessed June 10, 2020. |
|  |  | Residential income per capita | 2018 five-year estimate | United States Census Bureau, American Community Survey | USCB. (2021). American Community Survey. <https://www.census.gov/data.html>. Accessed June 10, 2020. |
|  |  | Percent of residents with Supplemental Nutrition Assistance Program (SNAP) benefits | 2017 | New Jersey Department of Community Affairs (NJDCA) | NJDCA. (2020). The 2017 Municipal Revitalization Index. [https://nj.gov/dca](https://nj.gov/dca/). Accessed December 9, 2020. |
|  |  | Local government spending in health, safety, welfare, housing, community engagement, & development | 2017 | United States Census Bureau | USCB. (2021). 2017 State and Local Government Finance Historical Datasets and Tables. <https://www.census.gov/data/datasets/2017/econ/local/public-use-datasets.html>. Accessed December 21, 2020. |
| Note. Source: Williams LD, et al. (2023), *Journal of Urban Health*. Reprinted with permission. | | | | | |
